# Machine-learning based patient classification using Hepatitis B virus full-length genome quasispecies from Asian and European cohorts

**DOI:** 10.1038/s41598-019-55445-8

**Published:** 2019-12-11

**Authors:** Alan J. Mueller-Breckenridge, Fernando Garcia-Alcalde, Steffen Wildum, Saskia L. Smits, Robert A. de Man, Margo J. H. van Campenhout, Willem P. Brouwer, Jianjun Niu, John A. T. Young, Isabel Najera, Lina Zhu, Daitze Wu, Tomas Racek, Gadissa Bedada Hundie, Yong Lin, Charles A. Boucher, David van de Vijver, Bart L. Haagmans

**Affiliations:** 1 Roche Innovation Centre, Basel, Switzerland F. Hoffmann-La Roche AG, Grenzacherstrasse 124, CH-4070 Basel, Switzerland; 2 Department of Viroscience, Erasmus Medical Center, Rotterdam, ‘s-Gravendijkwal 230, 3015 CE Rotterdam, Netherlands; 3 Zhongshan Hospital Xiamen University, 205 Hubin S Rd, Siming Qu, Xiamen, 361000 Fujian China; 4 Department of Gastroenterology and Hepatology, Erasmus Medical Center, Rotterdam, The Netherlands; 5 Roche Innovation Centre Shanghai, Roche R&D Centre (China) Ltd., 720 Cai Lun Road, Pudong, Shanghai 201203 China

**Keywords:** Machine learning, Viral infection

## Abstract

Chronic infection with Hepatitis B virus (HBV) is a major risk factor for the development of advanced liver disease including fibrosis, cirrhosis, and hepatocellular carcinoma (HCC). The relative contribution of virological factors to disease progression has not been fully defined and tools aiding the deconvolution of complex patient virus profiles is an unmet clinical need. Variable viral mutant signatures develop within individual patients due to the low-fidelity replication of the viral polymerase creating ‘quasispecies’ populations. Here we present the first comprehensive survey of the diversity of HBV quasispecies through ultra-deep sequencing of the complete HBV genome across two distinct European and Asian patient populations. Seroconversion to the HBV e antigen (HBeAg) represents a critical clinical waymark in infected individuals. Using a machine learning approach, a model was developed to determine the viral variants that accurately classify HBeAg status. Serial surveys of patient quasispecies populations and advanced analytics will facilitate clinical decision support for chronic HBV infection and direct therapeutic strategies through improved patient stratification.

## Introduction

HBV is a relaxed circular, partially double-stranded DNA virus, with a unique genomic organization (four overlapping reading frames encoding 7 proteins in 3.2 kb) and replication mechanism. The low-fidelity reverse transcriptase (1.45-7.9e-05 per site/year - non-synonymous *vs* synonymous mutations^1^) and the high replication rate (1e12 virions/day) means that a single progeny virus may not be identical to the parent genome in a single infected cell^2^. This generates a viral ecosystem consisting of mutant swarms or ‘quasispecies’, a population of genetically distinct, but closely related viral variants^3–5^. HBV quasispecies, therefore, comprise a spectrum of viral variants with differing fitness, which allows for the rapid adaptation to selective pressures including host immune factors and antiviral agents^6,7^. Consequently, HBV variants have an impact (if positively selected to sufficient abundance) on disease pathogenesis, clinical progression, and response to therapeutic interventions^8–11^. Frequently reported estimates for infection prevalence (4%), absolute number of infected individuals (257 million), and annual mortality (887000) demonstrate the large global burden of disease attributable to HBV^12,13^. Together with hepatitis C virus (HCV), viral hepatitis represents the leading cause of hepatocellular carcinoma^14,15^.

HBV e antigen is a secreted precore protein of Hepatitis B virus with an unclear viral function^16^, but with high sequence conservation across hepadenaviruses^17^. Seroconversion to this antigen and subsequent antigen loss (HBeAg negative status, anti-HBe positive phase) is associated with reduced viral replication and diminished long-term complications, for example, the risk of hepatocellular carcinoma^18^. Progression to seroconversion, either spontaneous or treatment-induced, may take years; contemporary studies have demonstrated differential rates of seroconversion associated with the duration and type of therapeutic regimen in addition to individual patient clinical profiles^19,20^. All virological factors associated with HbeAg seroconversion have not been elucidated and deeper knowledge of these may aid the definition of patients likely to seroconvert and direct appropriate therapeutic strategies.

In addition to HBV DNA load, ALT levels, HBV surface antigen (HBsAg), the loss of plasma HBeAg and seroconversion to HBeAg represent valuable clinical waymarks of a functional cure^21^. The plasma levels of these markers are used as proxies of viral activity in the liver; inferences about the spectrum of viral quasispecies in plasma reflecting those in hepatocytes has not been qualitatively assessed. Furthermore, HBeAg negative patients may have ongoing complex clinical profiles the virological basis of which has not been established.

The progression of chronic hepatitis B is related to the prevailing viral and host immune activities. The tolerogenic activity of HBeAg facilitates the establishment of HBV infection *in vivo* - this tolerance to infection is lost during the anti-HBe positive phase and immune-escape HBeAg-negative mutants may be selected^22^. Increased sequence diversity, associated with stochastic mutations, can result in changes in immune tolerance and reactivity with concomitant changes in selection pressure and evolution of the virus. The dynamic interplay and drivers of viral evolution are complex and difficult to resolve linearly; the use of advanced analytic tools may assist in the deconvolution of the prevailing virological factors that contribute to serocoversion.

Many studies have disclosed variants with statistical associations with clinical metrics in different regions of the HBV genome^23–26^, but associations between all variants in all HBV coding regions and clinical parameters has not been established. For example, the nG1896A (W28*) mutation in the precore and nA1762T/nG1764A double mutation in the basal core promoter have temporal correlations with seroconversion and have been shown to be associated with different clinical courses^3,11^. Next generation sequencing (NGS) technologies are optimal for uncovering the spectra of variants that exist within an infected individual and in the wider population for disease surveillance and healthcare planning^27^. Ultra-deep NGS enables the detection of viral variants at low allele frequency with much greater sensitivity and confidence. To date most studies have focused on particular regions of the HBV genome, e.g. HBs major hydrophilic region (MHR)^28^, core^29^, and reverse transcriptase (RT)^30^ or on whole genome sequencing^9^ at low coverage and, most recently, on single virions^31^. Here we present the first comprehensive survey of the diversity HBV quasispecies through ultra-deep sequencing of the complete HBV genome across two distinct patient populations and, in addition, explore quasispecies diversity between plasma and liver samples.

**Figure 1:**
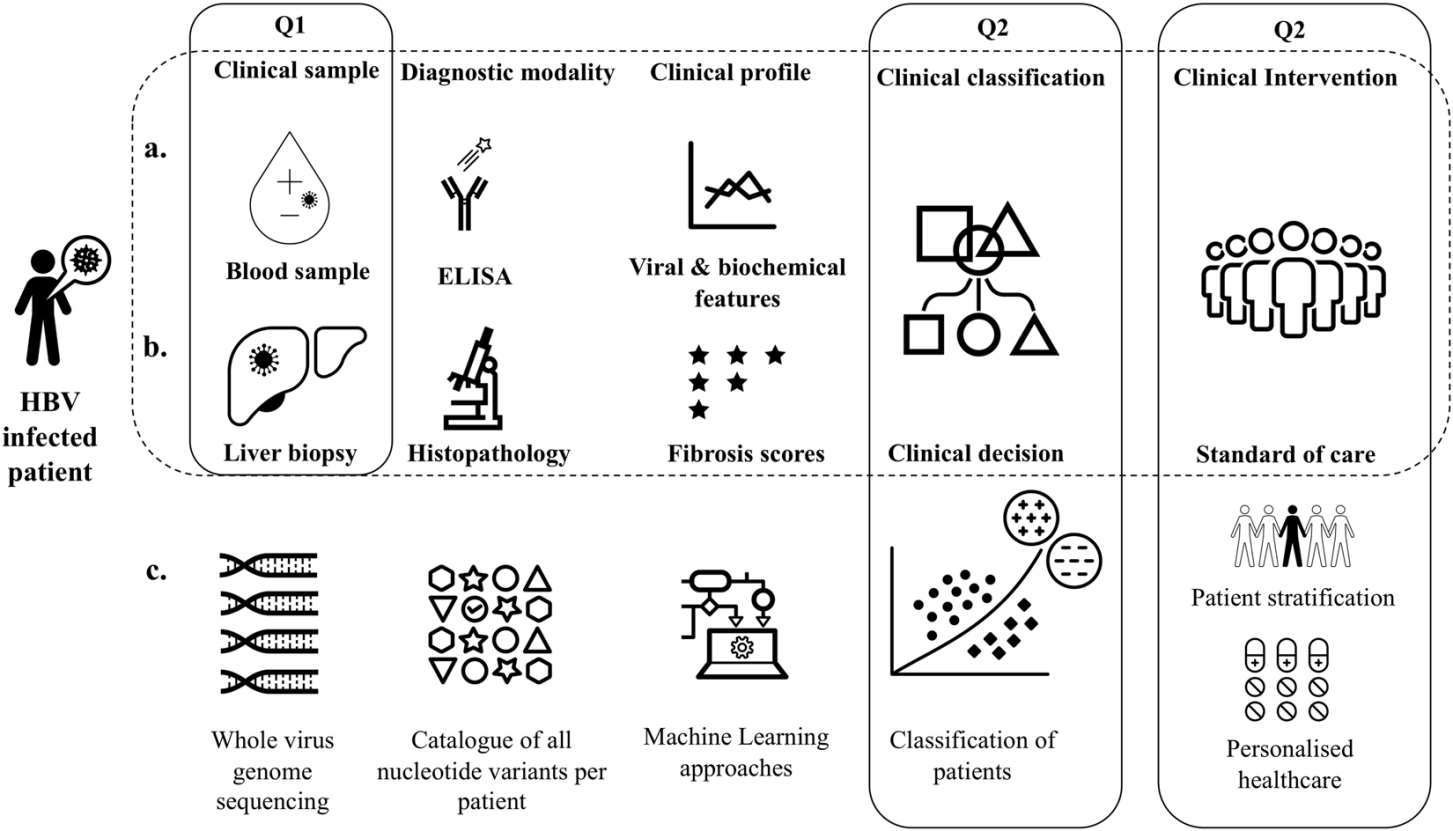
Study summary and motivation. Patients infected with HBV have complex and dynamic clinical profiles. The diagnostic and clinical decision paradigm (dashed box) in HBV infected patients involves classification by plasma markers of viral activity (**a**) and biochemical and histopathological (**b**) evidence of liver damage. This approach defines patients broadly into four classes that will inform the clinical decision for standard-of-care including the use of interferon and/or nucleoside analogues. Using virus whole genome sequencing to catalogue all nucleotide variants occurring at >1% machine learning approaches are explored to determine whether classification of HBeAg status could be recapitulated from a diverse patient population, extend our understanding of the virological factors associated with HbeAg status and evaluate whether this type of approach may be extended to novel markers of clinical status that will inform clinical decision making for stratification of patients in clinical trials and in the appropriate patient selection for use of next-generation treatment modalities for HBV. The study sought to answer three questions (solid boxes): Q1 - that plasma HBV quasispecies profiles were representative of those in the liver; Q2 – that machine learning approaches could accurately recapitulate classification by a routinely used clinical marker; and Q3 – whether this approach has wider utility in clinical decision support and the deconvolution of complex clinical history.

The use of big data advanced analytics is an emergent field with potential for extensive application in healthcare^32,33^ including applications where there is disease and treatment heterogeneity and for prescriptive analytics (precision medicine and clinical decision support). The current paradigm for classification of HBV infected patients utilises a series of virological and biochemical factors to infer viral activity and liver damage, as well as histopathological analysis of liver biopsies for fibrosis scoring (Fig. 1). In complex viral disease profiles, where single laboratory tests may not provide sufficient insight into the clinical history and progression of a patient advanced analytic techniques, including machine learning, may unlock insights into host and pathogen factors that may represent novel biomarkers for prognostics and therapeutic strategies. Consideration of advanced analytics applied to complex disease of virological origin has not been extensively evaluated - this study sought to investigate the feasibility of combining big data and advanced analytics to drive clinical insight in chronic hepatitis B infection.

Machine Learning (ML) encompasses a field of data-driven techniques for the study and construction of predictive algorithms that allow classification of factors/classes or estimation of quantitative traits from complex, multi-dimensional data (hundreds to thousands of co-variates) without *a priori* models^34^. In essence, ML approaches identify patterns revealing novel relationships between covariates or allow inferences to be made about future events. We have applied a random forest machine learning approach to classify the HBeAg status of untreated patients with chronic HBV infection using the standard HBeAg diagnostic test as a benchmark. Using the allele frequency of HBV variants we identify novel associations between viral variants and HBeAg status. Our analysis demonstrates a proof-of-concept of the utility of machine learning approaches to classify HBV infected patients and offers the prospect of exploring additional markers for therapeutic decision making and prognostic support.

## Methods

### Patients and samples

Patient samples were derived from two retrospective cohorts representing a single point in time for each patient; the first, a Western European cohort defined as Dataset A, the second, a Chinese cohort, defined as Dataset B, Table 1. In both cases the collection of patient samples was undertaken with informed and written consent and in accordance with the Declaration of Helsinki. Metadata (demographic and clinical data) was anonymized at point of collection and all data analysis was undertaken blinded and without access to patient identification keys. Projects were reviewed and authorized by the respective ethical review boards (Dataset A: Erasmus Medical Centre, Rotterdam, The Netherlands; Dataset B: Ethics Committee of Xiamen Centre for Disease Control and Prevention). Dataset A was derived from plasma samples (n = 182) consecutively collected from chronic HBV patients between 1985 and 2012 and stored at the Erasmus University Medical Centre, Rotterdam, The Netherlands. Dataset B plasma samples (n = 207 samples) were collected from patients with chronic HBV infection attending the Zhongshan Hospital, Xiamen University, China between 2013 and 2016. Patient inclusion criteria in Dataset B related to age (20 to 79 years old) and HBV DNA levels (≥10^7^ copies/ml).

**Table 1:** Summary descriptors for patients included in the study.

Description	Dataset A	Dataset B
**Gender**		
Female	55	67
Male	127	140
Duration of infection (years)	33.2 (±13.2)	Unknown*
Age at inclusion (years)	35.5 (±13.5)	30.6 (±7.2)
HBV DNA load (log10 copies/mL)	6.74 (±1.96)	7.75 (±0.46)
AST (IU/mL)	53.1 (±33.07)	134.7 (±125.3)
ALT (IU/mL)	87.8 (±80.02)	265.4 (±272.9)
**HBeAg status**		
negative	85	26
positive	97	181
**Treatment**		
naive	182	170
established	0	37

Datasets A and B represent the European and Asian cohorts respectively. Pertinent demographic, virological and biochemical data are provided. Continuous data is provided as the mean and standard deviation for the appropriate dataset. The duration of infection was not available for the Asian cohort as a result of the nature of standard presentation of patients in the recruiting clinic. *Estimated duration of infection unavailable.

### Diagnosis

For Dataset A serum HBeAg was quantified in samples taken at baseline using the Elecsys® HBeAg assay (Roche Diagnostics, range 0.2–100 IU/ml). HBV infection of patients was assessed using commercial enzyme-linked immunosorbent assay kits. Paired liver biopsies (collection procedure was undertaken as previously described^35^) and plasma were obtained from ten patients in accordance with the approved study protocol. For Dataset B the HBV infection of patients was confirmed by using commercial enzyme-linked immunosorbent assay kits (Wantai BioPharm, Beijing, China) and quantified by using real-time fluorescence quantitative PCR. Patient liver biochemistry and virological metrics (serum qualitative HBsAg, anti-HBs, HBeAg, anti-HBeAg, HBV viral load) at baseline was obtained from the respective electronic medical records.

### Deep-sequencing

The ultra-deep sequencing of HBV samples was conducted by DDL Diagnostic Laboratory (Dataset A, Rijswijk, Netherlands) and by Guangzhou Kingmed Diagnostics (Dataset B, Guangzhou, China). Multiplexed paired-end sequencing was performed on the Illumina MiSeq platform using the MiSeq v2 sequencing kit with 300 cycles for both sample populations. Demultiplexed FASTQ files were generated as an output. A complete description of workflow is provided in Supplementary Materials and Methods.

### Data analysis pipeline

The sequencing analysis pipeline was implemented in *python* (Python 2.7, 12.05.2015). All further data analysis, machine learning, phylogenetic and statistical analysis (including Shannon entropy and majority voting analysis) and graphical plot generation was performed in *R* (Release 3.4.1, 30.06.2017), The R Foundation for Statistical Computing) – a full list of software packages and methods are provided in Supplementary Materials and Methods.

### Sequencing analysis pipeline

Nucleotide sequences for reference genotypes were obtained from NCBI (https://www.ncbi.nlm.nih.gov/nuccore) with the following accessions: genotype A (AF090842); B (AB033554); C (AB033556); D (AF121240); and E (AB032431). FASTQ files were obtained from Illumina sequencing platform output for all samples. A full description of the sequencing analysis pipeline is provided in Supplementary Materials and Methods and Supplementary Fig. 2.

### Machine learning

To classify HBeAg status from viral mutant signatures for both datasets only samples from untreated patients were used (n = 182 Dataset A; n = 170 Dataset B) to limit effects on sample variant profile attributable to treatment methods. The input to the machine learning was a matrix of viral variant allele frequencies (0.01–0.99) and the associated HBeAg status (‘positive’ or ‘negative’) as defined by standard diagnostic tests. A random forest machine learning approach was employed to establish the variants that best classified HBeAg status. A series of test and training partitions and cross-validation steps were undertaken to optimize the model before testing against independent data excluded from model generation. Comparison of random forest models was based upon the following metrics: accuracy, balanced accuracy, sensitivity, specificity, and kappa values. For the combined dataset (Datasets A and B: n = 352 samples and n = 5533 unique variants) variants with near-zero variance were excluded leaving n = 432 variants. Full descriptions of the machine learning approach are provided in Supplementary Materials and Methods and Supplementary Fig. 3.

## Results

### Comprehensive survey of HBV variants

#### Supplementary tablers liver HBV variants

**Figure 2:**
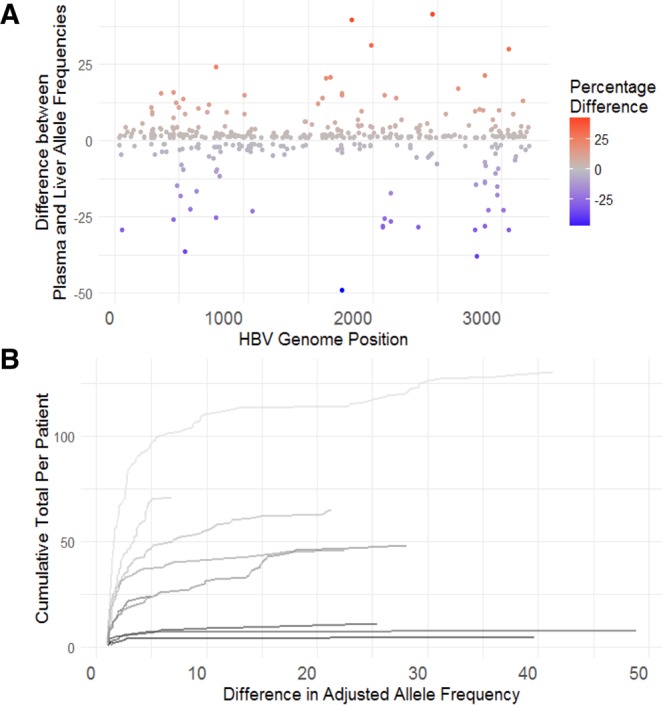
Allele frequency differences between liver and plasma. (**A**) Percentage differences (y-axis) in allele frequency between variants found more abundantly in the plasma (red points, positive values) or liver (blue points, negative values); HBV genome nucleotide positions defined by the x-axis. (**B**) Cumulative frequency plot for difference (>1%) in allele frequency for variants in n = 10 paired liver and plasma samples - each trace represents the running total trace for the frequency of variants with a defined difference in a allele frequency between liver and plasma samples. The majority of variants found in liver and plasma only show small differences in the adjusted allele frequency (1–2 percentage points) as demonstrated by the steep rise of traces in most patients. Few variants (n = 56) show differences >10% across all samples.

To investigate whether plasma samples sufficiently represent the hepatic quasispecies population paired liver biopsy and plasma samples were collected as part of Dataset A (n = 10). The frequency of HBV variants found in either the liver or plasma were compared to define systematic differences in the quasispecies populations. The majority of variants demonstrated small differences in allele frequency (1–2%), as shown in Fig. 2A. A small number of variants (n = 56) demonstrated a difference of 10% or greater. These differences between plasma and liver populations were found to be patient-associated, i.e. the majority of patients demonstrated only small differences in allele frequency between the tissues (Fig. 2B), whilst one patient accounted for the majority of variants showing large differences in allele frequency (Supplementary Table 2a,b).

#### Quasispecies diversity arises from rare and unique viral variants

**Figure 3:**
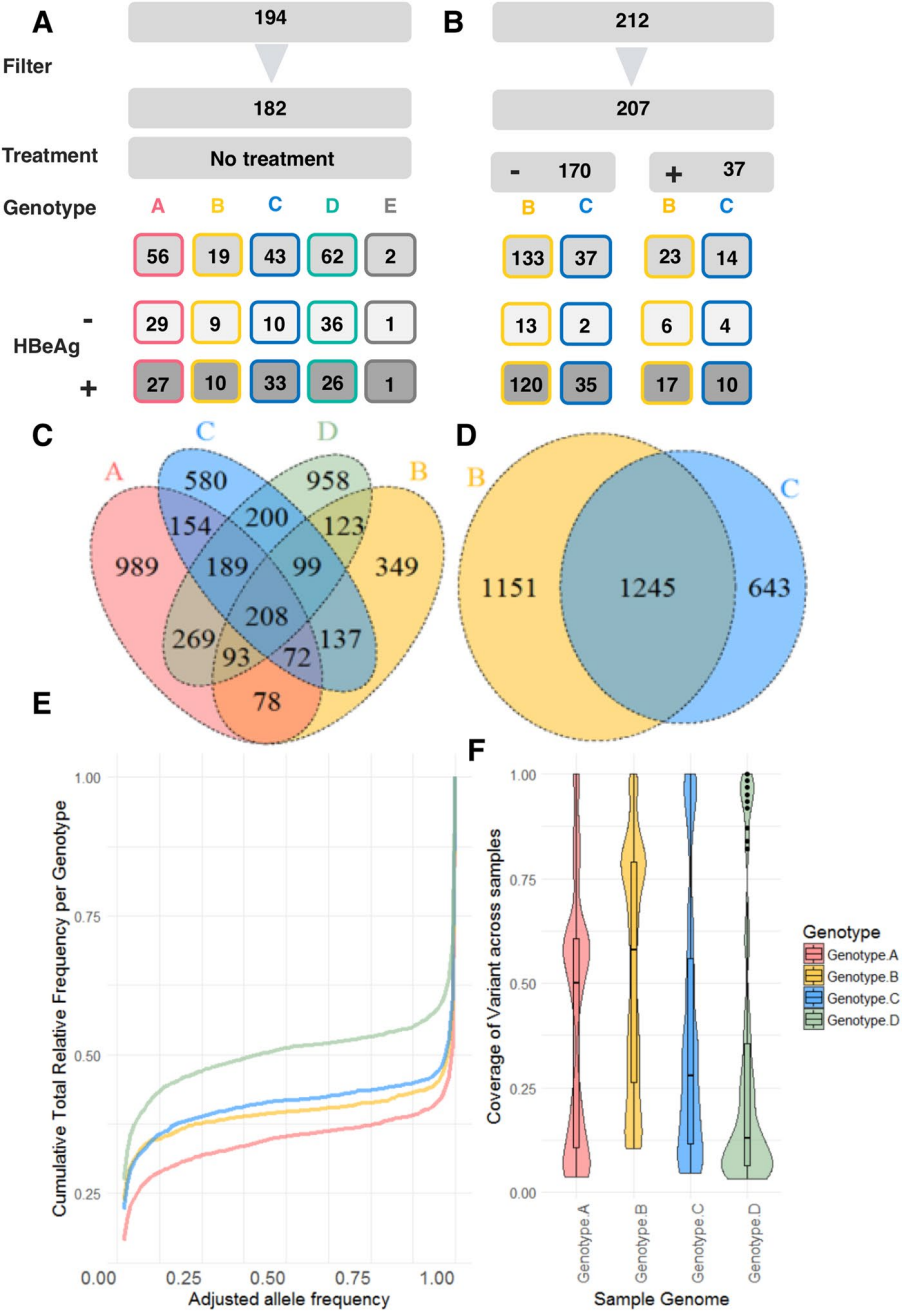
Graphical overview of the distribution of samples from both datasets. Dataset A (**A**) consisted of n = 182 patients; Dataset B (**B**) was derived from n = 207 patients. The overlap of variants identified between different genotypes for Dataset A (**C**, Venn plot not scaled) demonstrated n = 208 variants common to genotypes **A**–**D**). In Dataset B genotypes B and C shared a larger number of variants (**D**). Frequency distribution - the majority of variants were either rare or uncommon with an adjusted allele frequency above the limit of detection (1%) or represented the most prevalent allele in the samples (**E**). (**F**) Violin plots show the distribution of variants within each genotype with frequency of variant occurrence within a genotype presented as the coverage (count/number of samples) where 1 represents a variant present in all samples of the same genotype (upper limits of the plot). Variants at the lower limits of the plots were rare and unique mutations and represented the sequence diversity in a genotype.

Dataset A (n = 182 patients) represented a cohort of diverse ethnic background from Western Europe. The dataset consisted of five HBV genotypes (A-E); all samples were obtained from untreated patients. A graphical overview of Dataset A is presented in Fig. 3A. Variants with an adjusted allele frequency greater than 1% were used for analysis. A total of 4615 variants were detected above this threshold and used as features to predict HBeAg status (Fig. 3C). In the absence of HBsAg levels it was not possible to classify the clinical status of these patients by EASL guidelines^21^. Dataset B was derived exclusively from an ethnically Chinese (Han) patient cohort. After filtering Dataset B represented n = 207 patients of whom n = 170 were treatment naïve; a further n = 37 received either treatment with a nucleoside analogue or interferon therapy (Fig. 3B). Using the EASL (European Association for the Study of the Liver) guidelines all samples from this cohort were classed as ‘chronic hepatitis’. Dataset B was comprised of genotypes B and C; a total of n = 3039 variants were defined at >1% frequency with n = 1245 variants common to both genotypes (Fig. 3D). The intersection of genotypes B and C found n = 1640 variants common to both datasets (Jaccard Index = 0.45).

#### Viral variant heterogeneity differs with genotype

The average number of variants *per* patient and *per* genotype was considered for Dataset A (genotypes A-E represented). In general, within a viral genotype most variants were found only once across all the patients, Fig. 3E and Supplementary Fig. 4. To consider the overlap between patients across genotypes and define ‘genotype-associated’ variants we considered variants occurring at least twice within a genotype (Fig. 3F). Genotype D demonstrated the greatest sequence diversity with low variant coverage, i.e. few variants found consistently in all patient samples. Significant differences in Shannon entropy between HBeAg status groups was also noted (Supplementary Materials & methods; Supplementary Figs. 5–7)

#### Variants associated with drug resistance present in untreated patients

Low frequency mutations aid viral adaptation to selection pressures and so are particularly relevant to development of drug resistance and treatment failure^36^. Genomic variants in the reverse transcriptase/polymerase (RT/POL) gene, leading to amino acid substitutions conferring single and multi-drug resistance, were found in plasma samples of untreated patients in both datasets (Table 2). Drug-resistance associated mutations were found in 15–17% of patient samples from genotype B and D in dataset A. Patients infected with HBV genotype C demonstrated the most frequent resistance-associated variants in Dataset A (26%) and Dataset B (16% untreated; 29% treated).

**Table 2:** Drug Resistance-Associated Mutations and associated allele frequencies.

Dataset	Gene	Amino Acid alteration	Nucleotide	Allele Frequency	Absolute Number of Samples	Genotype	Resistance association
A	RT	Leu80Ile	367T > A	0.72	1/19	B	L
A	RT	Val84Met	379G > A	0.13–0.16	1/62	D	A
					1/43	C	
A	RT	Asn238Asp	841A > G	0.02–0.99	6/43	C	A/L
				0.17	1/56	A	
A	RT	Ala181Thr	670G > A	0.02–0.06	3/62	D	A/L/Tb/Tf
		Ala181Val	671C > T	0.02	1/62		
A	RT	Met204Ile	741G > T	<0.02	3/62	D	L/Tb ± E,A
				0.01	1/43	C	
				0.02	1/56	A	
			741G > T/C	0.01/0.73	2/19	B	
A	RT	Val214Ala	770T > C	0.02–0.05	4/62	D	A/Tf
				0.34	1/56	A	
				0.02/0.05	2/43	C	
		Val214Glu	770T > A	0.08	1/43	C	
B	RT	Val173Leu	646G > C/T	0.09/0.04	1/37	C	L/E
			646G > C	0.01	1/133	B	
B	RT	Ala181Thr	670G > A	0.01	1/133	B	A/L/Tb/Tf
				0.24	1/23^A^	B	
				0.02	1/14^E^	C	
B	RT	Ala194Thr	709G > A	0.04	1/133	B	Tf
B	RT	Met204Ile	741G > T	0.98	1/23^A^	B	L/Tb/Tf
B	RT	Val214Ala	770T > C	0.04	1/14^E^	C	A/Tf
				0.02	1/14^IFN^	C	
				0.03	1/133	B	
				0.02/0.04	2/37	C	
		Val214Glu	770T > A	0.8	1/14^IFN^	C	
B	RT	Asn238Asp	841A > G	0.02–0.03	3/37	C	A/L

In Dataset B patients receiving treatments are highlighted as a superscript letter in column 5 (absolute numbers of patients). Amino acid changes and related nucleotide positions are provided. The allele frequency for a mutation relative to the reference genomes is provided as a range where the mutation was found in more than two patients. In some individuals more than one mutation is present at the same locus. Abbreviations for therapeutics: **A** – Adefovir; **E** – Entecavir; **IFN** – Interferon; **L** – Lamivudine; **Tf** – Tenofovir; **Tb** – Telbivudine.

#### Phylogenetic analysis recapitulates genotype groups across cohorts

**Figure 4:**
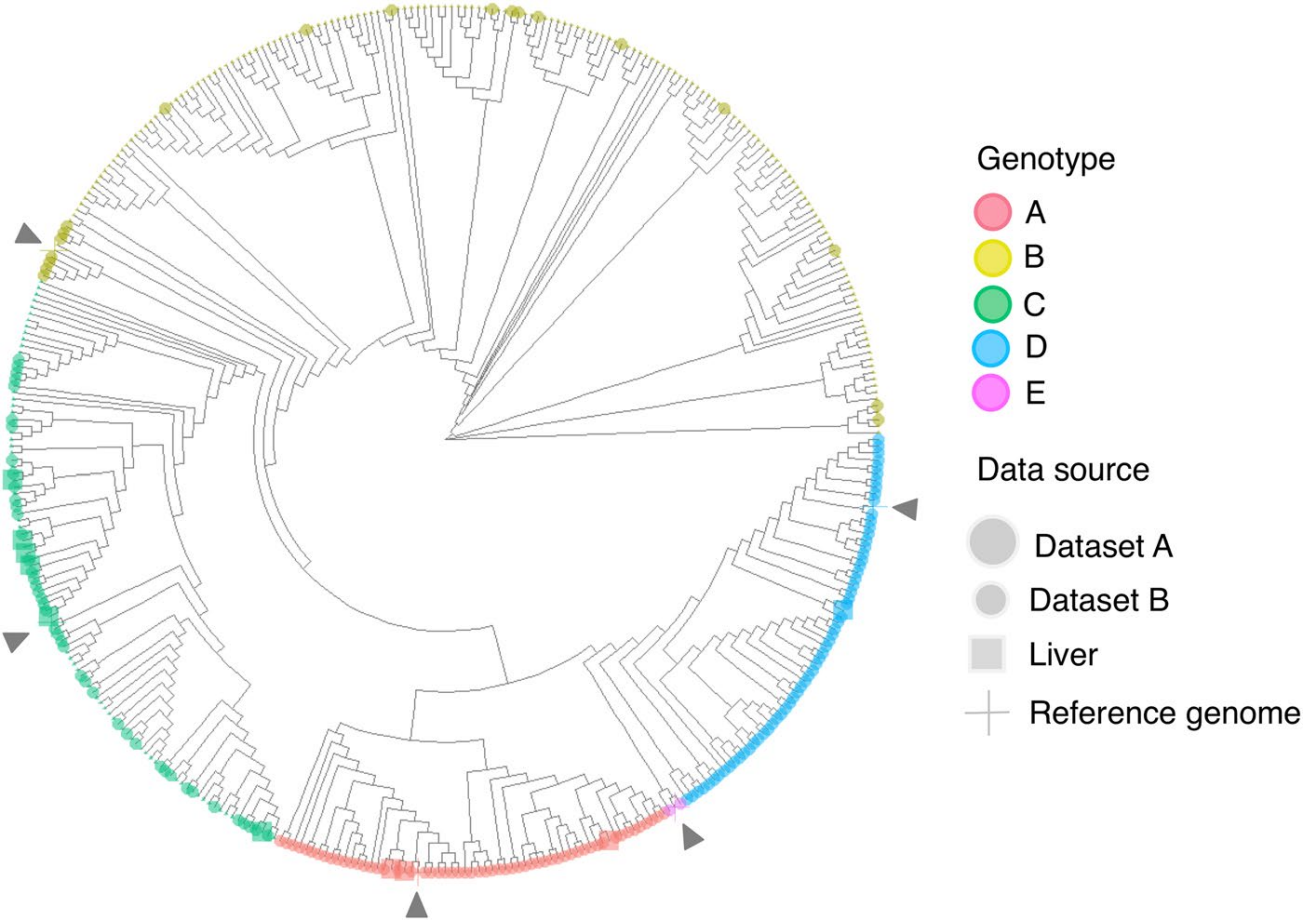
Circular cladogram based on n = 404 whole genome consensus nucleotide sequences. Phylogenetic analysis on sequences from n = 192 Dataset A (including 10 liver sample sequences), n = 207 Dataset B, and n = 5 reference strains. Figure key indicates genotype (by colour) and data source (by size and shape). Reference strains are defined by a ‘+’ and highlighted with dark-coloured arrow heads. Samples derived from n = 10 liver biopsies in Dataset A are defined by squares.

Consensus whole genome nucleotide sequences were gathered for each patient plasma sample for each dataset (Dataset A, n = 182; Dataset B, n = 207), paired plasma and liver samples (n = 10) variant profiles if present (total samples, n = 399), and five reference genomes (genotypes A-E) for phylogenetic analysis. Genotypes B and C grouped together in the appropriate clades, Fig. 4, regardless of geographical origin. The remaining Dataset A samples from genotypes A, D, and E also grouped together in their appropriate clades alongside the reference genomes used for mapping of sequencing reads. Despite the extensive sequence diversity recorded and clinical heterogeneity phylogenetic clades resolved entirely by genotype across the two datasets.

### Machine learning defines novel viral variants classifying HBeAg status

#### Defining classifier variants for HBeAg status

**Figure 5:**
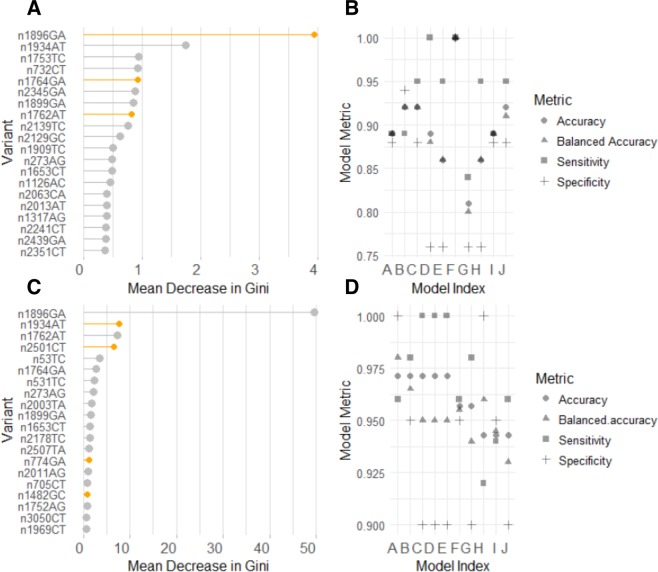
Highest ranking variants in machine learning model. (**A**,**C**) –Plots of the mean decrease in Gini Index for top 20 variables contributing to the best-performing models for Dataset A (5A) and combined data (5C); (**B**,**D**) – Plots of model metrics from each of ten models developed from Dataset A (5B) or combined dataset (Dataset A and B) (5D). Variants nomenclature: e.g. n1896GA represents a G > A mutation at nucleotide position 1896. (**A**) Gini plot of variant importance based on n = 4215 variants from Dataset A data associated with Model F (**B**). Model achieves a balanced accuracy of 1 with defined data partitioning. Variants highlighted in orange represent the stop-gain mutation (G1896A) and the two mutations of the basal core promoter (G1764A and A1762T). (**C**) Gini plot for top 20 variants contributing to the model developed from n = 432 variants to predict HBeAg status combining samples from untreated patients in Datasets A and B. The best model accuracy found in Model A (balanced accuracy = 0.98), (**D**). Variants depicted in grey (4C) were common to both datasets; those represented in orange were the mutations defined in Dataset A alone and are genotype-associated.

Machine learning was used to define patterns of viral variant allele frequencies that demonstrated a strong association with HBeAg status and would act as robust classifiers and uncover novel virological factors. A model with a balanced accuracy of 1 (Accuracy, Sensitivity, and Specificity = 1) was found using test data from Dataset A (range balanced accuracy 0.8–1), Fig. 5A,B, although the relative contribution of each variant to classification accuracy was small. The highest-ranking variables contributing to this model included known pre-core and basal core promotor mutants (n1896GA, n1934AT, n1753TC). In general, the variants with greatest relevance to the model were found in the precore/core region, with some variants found in the HBsAg, X or RT/POL genes. The majority of variants were mis-sense, with nG1896A and nC2351T defined as stop gains.

**Figure 6:**
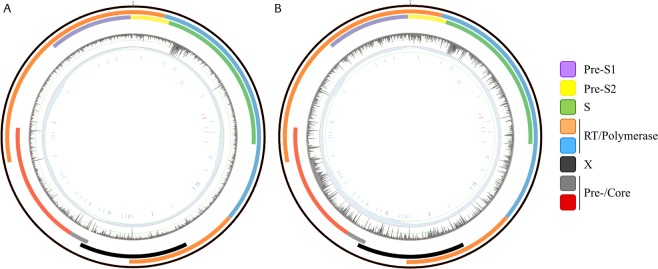
Circos plots depict distribution of high-ranking variants across HBV genome. Dataset A genotypes B and C. From outer to inner layer: Circos plot representing the HBV genome (layer 1) genotypes B and C (3215 nucleotides); layer 2–4: representation of the relative position of the transcribed genes; layer 5: mean entropy for each nucleotide position (0–0.15) with y/vertical-axis marks representing the following: 0, 0.03, 0.06 and 0.09 (outer to inner); layer 6: Read coverage *per* nucleotide position (maximum 60000) – vertical axis, (i) 5000 (red), (ii) 10000 (green), (iii) 20000, iv) 30000; layer 7: individual nucleotide position for the top 50 ranked variants contributing to the generic machine learning model for prediction of HBeAg status; layer 8: nucleotide positions of variants, found in untreated patients, associated with resistance to therapeutics. Arrows define the approximate location of the proprietary primers used. (**B)** Circos plot generated from dataset B follows the same topology as plot A. Gene annotation is provided by the colour key.

Viral variant allele frequencies from Datasets A and B were combined (n = 352 samples and n = 2119 common variants). This represented a low overlap of shared variants across datasets (Jaccard index = 0.38). Random forest classification of HBeAg status utilised samples from treatment naïve patients from both cohorts with n = 100 HBeAg negative and n = 252 HBeAg positive samples. The most accurate models were achieved when variants with near-zero variance were excluded from combined dataset leaving n = 432 variants for machine learning. The best performing model had a balanced accuracy of 0.98 (accuracy = 0.97; sensitivity = 0.96; specificity = 1; kappa: 0.93) against test data derived from both data sets (0.9/0.1 ratio of train/test data), Fig. 5C,D. By combining the datasets the relative contribution of each variant to classification accuracy to improved and altered the ranking of the variants with the greatest contribution. The distribution of the top-ranking variants in the HBV genome is presented in Fig. 6A,B. Using this model to classify HBeAg status of the additional n = 37 samples (HBeAg status: n = 10 negative; n = 27 positive) from treated patients (not seen in the training of the model) was highly accurate (balanced accuracy = 1).

## Conclusions

Hepatitis B virus has a baseline mutation rate of 1–7 e-05 base mutations/year and this diversity is fundamental to an understanding of the associations with the course of clinical disease, mutational dynamics, evaluation of novel strains, susceptibility to therapeutic interventions and development of immunity^27^. We report the first study to undertake ultra-deep sequencing of the whole HBV genome in almost 400 patients from two distinct cohorts. Ultra-deep sequencing demonstrated that, in general, variants arising within a single patient represented unique or rare mis-sense mutations that occur at the level of detection for the platform used (>1%). A subset of variants were found exclusively or with high frequency within the distinct genotypes. Differences were evident between genotypes in terms of the heterogeneity of viral populations with genotype D samples revealing greater sequence diversity with many low frequency mutants within individuals and more variation between patients than genotype A; greater similarity was evident between genotypes B and C.

The quasispecies populations of paired liver and plasma samples were analysed in a small patient cohort (n = 10). Few variants demonstrated a differential allele frequency greater than 10 percentage points between liver and plasma; we concluded that plasma samples represented an adequate proxy for inferring the diversity in the viral quasispecies population in chronically infected patients. In work by Nishijima and colleagues^37^, to determine the correspondence of hepatic quasispecies with plasma populations in patients undergoing liver transplantation, no differences in Shannon entropy was found. Likewise, no significant differences have been found between multiple paired liver samples and plasma in HCV-infected patients^38^. Coffin, *et al*., reported location and disease phase-specific differences in variants^39^ although limited differences were found between liver, polymorphonuclear cells (PBMCs) and plasma over an extended period of analysis^10^. This corroboration of quasispecies populations in liver and plasma supports the further investigation of plasma biomarkers to evaluate the presence of disease-associated variants.

Contemporary sequencing studies have focused on particular regions of the HBV genome, e.g. HBs MHR^28^, core^29^, and RT^30^, or small whole genome sequencing studies^9^. Using ultra-deep sequencing, with high coverage, of the complete HBV genome we employed a random forest algorithm^40^, an ensemble method that builds ‘forests’ of binary decision trees to improve the classification accuracy of weak predictors, to define HBeAg status in patients across genotypes and demographics. Although machine learning approaches have been applied recently for virus data^41–43^ these methods have been applied sporadically to HBV^41,44–46^. The analysis presented here demonstrates the utility of unbiased, data-driven approaches to reveal novel aspects of HBV biology. Harnessing the power of two cohorts ML defined patterns of viral variant allele frequencies that accurately classified HBeAg status that was not possible from a single dataset; this model contained both variants with known temporal associations with HBeAg seroconversion, confirming the validity of the approach, and novel variants that aided the discrimination of HBeAg status that would not have been discovered by traditional statistical approaches.

HBe loss has a temporal correlation with elevated frequencies of a precore mutation at nG1896A and/or nG1764A/nA1764T basal core promotor mutations^47^ although these mutations are not present in all genotypes/sub-genotypes during seroconversion^22^. The ML models confirmed nG1896A as the most predictive mutation in classifying the HBeAg status of chronically infected HBV patients. Additionally, we note the strong contribution from nA1934T, nC2501T, nG1899A in HBeAg classification together with genotype-associated variants. The combined models included nC2501T, an intergenic variant found exclusively in genotype A in Dataset A at an allele frequency >0.91 in patients (n = 58) of both HBeAg classes. This variant is not associated with the six nucleotide insertion in genotype A. This highlights genotype specific discrimination in the combined model where the majority of samples were genotype B (n = 187). Similarly, the nA1934T precore/core variant was associated with genotype A, D and E samples and not found in genotype B and C samples in either dataset indicating another genotype partition in the combined model. The nA1934T mutation is reflected in a Thr12Ser amino acid substitution and is a mutation within the MHC class II restricted T-cell epitope (CD4 + T_h_ epitope 1–20) in the core protein^48,49^ known to be associated with clinical reactivation during lamivudine treatment^50^ and HCC^51^. This suggests that these variants contribute to partitioning of genotypes with subsequent classification of HBeAg status by the frequency of nG1896A, nG1899A, nG1764A/A1764T mutations.

This study identifies novel patterns of viral variants associated with existing HBeAg status, however, makes no inference about the mechanism by which this status was reached (no historical clinical data) or what the model means with respect to patient outcomes (data was not part of a longitudinal study). Equally, we cannot comment on patient profiles that are not represented in the study population, e.g. HBeAg negative inactive carriers with low HBV DNA loads. The ML model was formed on the known HBeAg status as defined by standard diagnostic techniques, i.e. the development of an alternative diagnostic test was not the intent of this study. Although we present a classification model with high discriminative accuracy this does not translate directly to changes in clinical practice and decision support. To make such a model applicable we require prospective studies with serial sampling to capture patients in the process of seroconversion and follow treatment groups. Further, such a model requires to be calibrated to the population of interest (e.g. our study uses two clinical cohorts with differing healthcare approaches) and be applied to a clear risk-sensitive decision point in the clinical setting (e.g. change in therapeutic regimen)^52^. We are, however, encouraged by the performance of the general model in discriminating HBeAg status in the n = 37 patients receiving treatment from the Dataset B cohort which served as an independent test group. By including a diverse sample population for feature-selection it was possible to establish a general model for HBeAg classification with broad relevance to the clinical population. Furthermore, we characterize the incidence of resistance-associated mutants in naïve patients; previous work has shown the presence of these mutants in naïve patients^30,53^ and our work further supports the future requirement for baseline sequencing of infected individuals to tailor therapeutic regimens.

The utility of ML approaches to clinical decision making in infectious diseases is not currently widely appreciated, however, the application of deep sequencing and ML analysis to identify data patterns could facilitate the targeting of specific therapeutic interventions to high risk groups, aid stratification of patients for more effective clinical trial design, link models to clinical decision support tools and, through incorporating patient demographic data, facilitate epidemiological and healthcare planning through a deeper understanding of the relationship between compound factors^27,32,33,54^. Our study demonstrates that plasma HBV quasispecies adequately represent the viral populations within hepatocytes and that these profiles, when interrogated with machine learning approaches, can recapitulate classification of patients by clinical marker status in addition to revealing novel biology.

## Supplementary information

Supplementary Materials and Methods
