# Supplementary material for: Machine-learning based patient classification using Hepatitis B virus full-length genome quasispecies from Asian and European cohorts
Source: Sci Rep. 2019 Dec 11;9:18892. doi: 10.1038/s41598-019-55445-8 (PMC6906359; doi:10.1038/s41598-019-55445-8)
Supplement: Supplementary file 1 — Supplementary Materials and Methods [file 41598_2019_55445_MOESM1_ESM.docx]

**Supplementary Data**

**Machine-learning based patient classification using Hepatitis B virus full-length genome quasispecies from Asian and European cohorts**

Alan J. Mueller-Breckenridge, Fernando Garcia-Alcalde, Steffen Wildum, Saskia L. Smits, Robert A. de Man, Margo J.H. van Campenhout, Willem P. Brouwer, Jianjun Niu, John A.T. Young, Isabel Najera, Lina Zhu, Daitze Wu, Tomas Racek, Gadissa Bedada Hundie, Yong Lin, Charles A. Boucher, David van de Vijver, Bart L. Haagmans.

**Methods**

Supplementary Methods make reference to two datasets: Dataset A – cohort of Western European patients and Dataset B, a cohort of exclusively Chinese patients.

*HBV DNA amplification*

Gene and fragment-specific amplification primers for Dataset B are provided in Supplementary Table 1 and provided as a schematic representation in Supplementary Figure 1.

*Phylogenetic analysis*

Multiple sequence alignment of n=404 sequences was performed using the Kalign[1] tool (<https://www.ebi.ac.uk/Tools/msa/kalign/>). Definition of genotype was verified using by CASTOR[2] (<http://castor.bioinfo.uqam.ca/>). Phylogenetic analysis was performed in *R* using the *phangorm*[3] package. Trees were plotted using *ggplot2*[4] and *ggtree*[5] packages in *R*.

*Shannon entropy*

In a set or string of symbols, for example a nucleotide sequence X ={AGGTCCGT}_n_, the probability of observing a particular symbol at the next position in the sequence is related to, i) the frequency of observing that symbol (based on previous sampling) and, ii) the number of possible outcomes (the number of different symbols).

Shannon’s metric of entropy [6] may be considered as quantifying the amount of ‘information’ stored in a variable, i.e. it quantifies the number of ‘bits’ that are required to encode each element of the set X and reflects the uncertainty associated with an outcome for a given random variable.

In a DNA sequence (a set of nucleotides, X) a single nucleotide position has a defined set of possible outcomes [A, C, G, or T] where each outcome has an associated probability [*p*_A_, *p*_C_, *p*_G_, and *p*_T_] calculated from the frequency of counts normalized to the coverage for each nucleotide position.

For a given variable X, the Shannon entropy H(X) is:

$\mathbf{H}\left( \mathbf{X} \right)\mathbf{= -}\sum_{\mathbf{i=0}}^{\mathbf{N-1}} \mathbf{p}_{\mathbf{i}}\mathbf{log}_{\mathbf{2}}\mathbf{p}_{\mathbf{i}}$ **Equation 1**

Where $p_{i}$ is the probability of observing a given symbol and i=(A,C,T,G). Each nucleotide position in the sequence has a distinct probability distribution quantified by the Shannon entropy – the entropy value could be considered as the uncertainty or unpredictability of defining a nucleic acid/residue in a sequence based upon the previous sampling.

Were each of the four nucleotide bases to occur at equal probability (0.25) at a single position the maximum Shannon entropy is:

H = *log (1/0.25) = log(4) = 2 bits*

Entropy is at its maximum when *p*_A_ = *p*_C_ = *p*_G_ = *p*_T_.

Entropy is zero when an event is certain to occur e.g. *p*_A_ = 1 and [*p*C, *p*G, *p*T] = 0.

In this case entropy = 0 where a single nucleotide position was fully conserved in multiple sequence alignment. To encode a highly variable region would require more bits because of the uncertainty at this position. Entropy only considers the probability of the specific event being observed - a statement about differential entropy describes inequality in the underlying probability distribution and does not give a biological meaning to the events *per se*.

In the context of sequencing hundreds of viral genomes in this study the Shannon entropy was normalized to the number of genomes evaluated as described in Nishijima, *et al*[7].

*Sequencing analysis pipeline:* An overview of the sequencing analysis pipeline is provided in Supplementary Figure 2. Quality control analysis of FASTQ files was performed using FASTQC (version v0.11.5, March 2016, https://www.bioinformatics.babraham.ac.uk/projects/fastqc/). Trimming and filtering of Illumina paired-end reads employed Trimmomatic (http://www.usadellab.org/cms/index.php?page=trimmomatic, version 0.39) [8], which also cleans reads for the presence of partial and complete Illumina adaptor and technical sequences and adaptive trimming of the reads with respect to average read quality (see Supplementary Table 3 for summary). The following parameters were used for trimming and filtering:

ILLUMINACLIP: Trimmomatic provided adapter file "TruSeq3-PE-2.fa"; HEADCROP: 15; CROP: 135; TRAILING: 3; SLIDINGWINDOW: window size:4: required quality: 15; MINLEN: 36.

Sample reads were aligned to the draft genome of the corresponding genotype using GSNAP with default parameters (<http://research-pub.gene.com/gmap/>, November 2016). Additional utilities for manipulating SAM/BAM and VCF files were provided from *samtools*[9], *picard* (http://broadinstitute.github.io/picard/) and *bcftools* (https://samtools.github.io/bcftools/) to obtain sorted alignment files in BAM format with non-duplicated uniquely mapped reads. Quality control of the BAM files was performed using x, v2.1.2 [10]. Nucleotide entropy was calculated using functions from *btctools* (now DiversiTools, <http://josephhughes.github.io/DiversiTools/>). Gene annotation to reference genome was performed with Exonerate (<https://www.ebi.ac.uk/about/vertebrate-genomics/software/exonerate> , v2.2.0) using the protein coding sequences defined in the NCBI database for each reference genome to produce GFF files with gene to protein annotations.

*Consensus genome generation*: In aligning to a reference sequence reads from any viral mutants that diverge significantly from the reference may be under-represented due to low alignment quality[11]. Variant inference was initiated on reads aligned to a reference genome, subsequently the consensus nucleotide at each position in the genome was defined with respect to the previous alignment, followed by iterative alignment of reads to the consensus viral genome using VirVarSeq (version 2015) [12] (<https://sourceforge.net/projects/virtools/files/>). This pipeline made use of Illumina quality scores in variant calling and so can reduced the incidence of false-positive calls and also increase coverage.

*Variant calling*: To accurately and reliably call rare variants from sequencing runs and distinguish from sequencing errors we incorporated LoFreq (lofreq_star 2.1.3.1, 2017) [13] , which utilises the *Q* values and read coverage for each base to derive a model of the sequencing error rate into the analysis pipeline. Annotation and prediction the coding effects of single nucleotide variants we implemented SnpEff (v4_3o, May 2017) [14].

*Machine Learning*

To classify HBV HBeAg status from viral mutant signatures for both datasets only treatment naïve samples were used (n=182 Dataset A; n=170 Dataset B) to limit effects on sample variant profile attributable to treatment methods. The input to the machine learning was a matrix of allele frequencies (0.1 – 0.99) and the associated HBeAg status (‘positive’ or ‘negative’). A random forest machine learning approach was employed to establish the variants that best classified HBeAg status. A series of test and training partitions and cross-validation steps were undertaken to optimize the model before testing against independent data unused for model generation. Exploration of data and feature selection was initially considered using principal component analysis (PCA). Two patients with high liver enzymes were defined as outliers by PCA and removed from Dataset A. Data preparation for machine learning required partitioning of the datasets into test and training groups (0.2:0.8 or 0.1:0.9 test to training data ratio). To ensure that test and training groups always contained representative proportions of the two classes balanced partitioning of data sets was undertaken in all cases. A nested partitioning and training approach was implemented to ensure predictive models were derived from multiple different subsets of the data (Supplementary Figure 3). Ten independent test/train partition sub-datasets, with resampling, were prepared such that any individual patient dataset could contribute to more than one sub-dataset. Each sub-dataset was then used for model training, optimisation, and validation. Resampling was performed on the training data set only (three repeats) with ten-fold cross-validation using the random forest algorithm as implemented in the *caret*[15] *R* package*.* A resampling index was created for *k*-folds cross-validation to ensure that class balance was maintained for within-training set resampling. A defined random number seed was set for all iterations; increasing the number of trees beyond 500 had no impact on model accuracy. In initial approaches machine learning was performed *ab initio* without variant filtering using the integrated feature selection tool; alternative feature selection processes did not out-perform the integrated feature selection method in *caret*. In all cases positive HBeAg status was used as the predictive class in model testing. Comparison of random forest models was based upon the following metrics: accuracy, balanced accuracy, sensitivity, specificity, and kappa values. For the combined dataset (integration of Datasets A and B) of n=353 samples and n=5533 unique variants selected features were those that did not have near-zero variance (n=432). These variant were selected using the *nearZeroVar()* function with defaults in ‘*caret*’ to define features that consist of only one unique value (zero variance predictors) or features that had few unique values relative to the total number of samples and features where the ratio of the frequency of the most common value to the frequency of the second most common value was large.

*Statistical analysis:* Deviations from the assumptions of parametric tests were assessed using Levene’s and Shapiro-Wilks tests. Where Gaussian distribution or equality of variance across groups was not met a Mann-Whitney-Wilcoxon test was applied in *R* (wilcox.test). False-discovery rate (FDR) corrections were applied to *p*-values across all pairwise tests for differences between groups with 0.05 defined as the threshold for hypothesis testing. To compare the overlap between groups of samples the Jaccard index, defined as the set intersection over the set union, was applied using the *SuperExactTest*[16] *R* package. A Jaccard index =1 would indicate complete overlap of the elements of both sets.

*Plots and figures*: All data figures were generated using *ggplot2*[4] and *R* base functions. Circos plots which were generated using Circos [17] v.0.69.

**Results**

*Viral entropy changes reflect HBeAg status*: Shannon entropy, a quantitative measure of information uncertainty, was defined for each nucleotide position of the HBV genome. The mean entropy was defined for each patient and differences in patient group means calculated for HBeAg status or genotype (Supplementary Figure 5), excluding genotype E. There was a significant difference (p<0.001, K-W test) between positive (entropy value, 0.016) and negative (0.023) HBeAg status for patients with HBV genotype A (n=56). The average entropy per nucleotide considered per gene or across the whole HBV genome demonstrated significant differences between HBeAg status for HBX (entropy value: 0.02 – negative, 0.016 – positive), HBsAg (0.026, 0.019), core (0.025, 0.016), and RT/POL (0.024, 0.017) in genotype A (Supplementary Figure 6) and genotype B, but not in genotypes C and D. In Dataset B there was no significant difference between mean per patient entropy when HBeAg status and genotype were considered, Supplementary Figure 5. An inverse relationship was noted in Dataset B for HBeAg status (Supplementary Figure 7), although this represented a smaller subset of the data.

**Supplementary Figures**


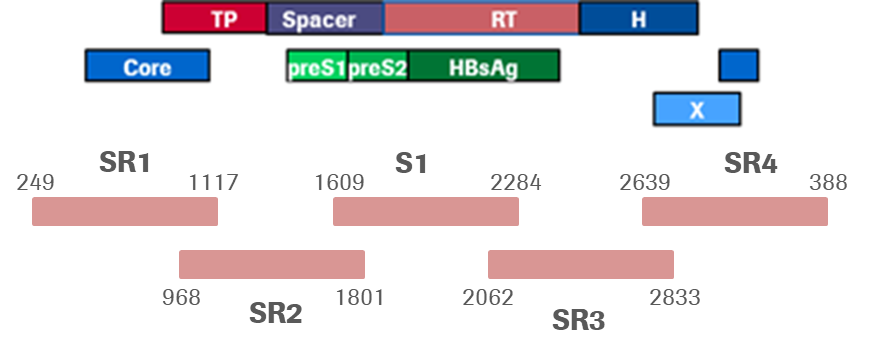


**Supplementary Figure 1**: Schematic representation of the HBV genome and the relative positions of the primers used for nested PCR amplification of viral DNA. Pool 1 consisted of primer pairs S1, SR2 and SR4; Pool 2 consisted of primer pairs for SR1 and SR3.

**Supplementary Figure 2**: Overview of the data analysis, manipulation and quality control methods used for the paired-end sequencing output for HBV genome. Sources and software are referred cited in the main text.


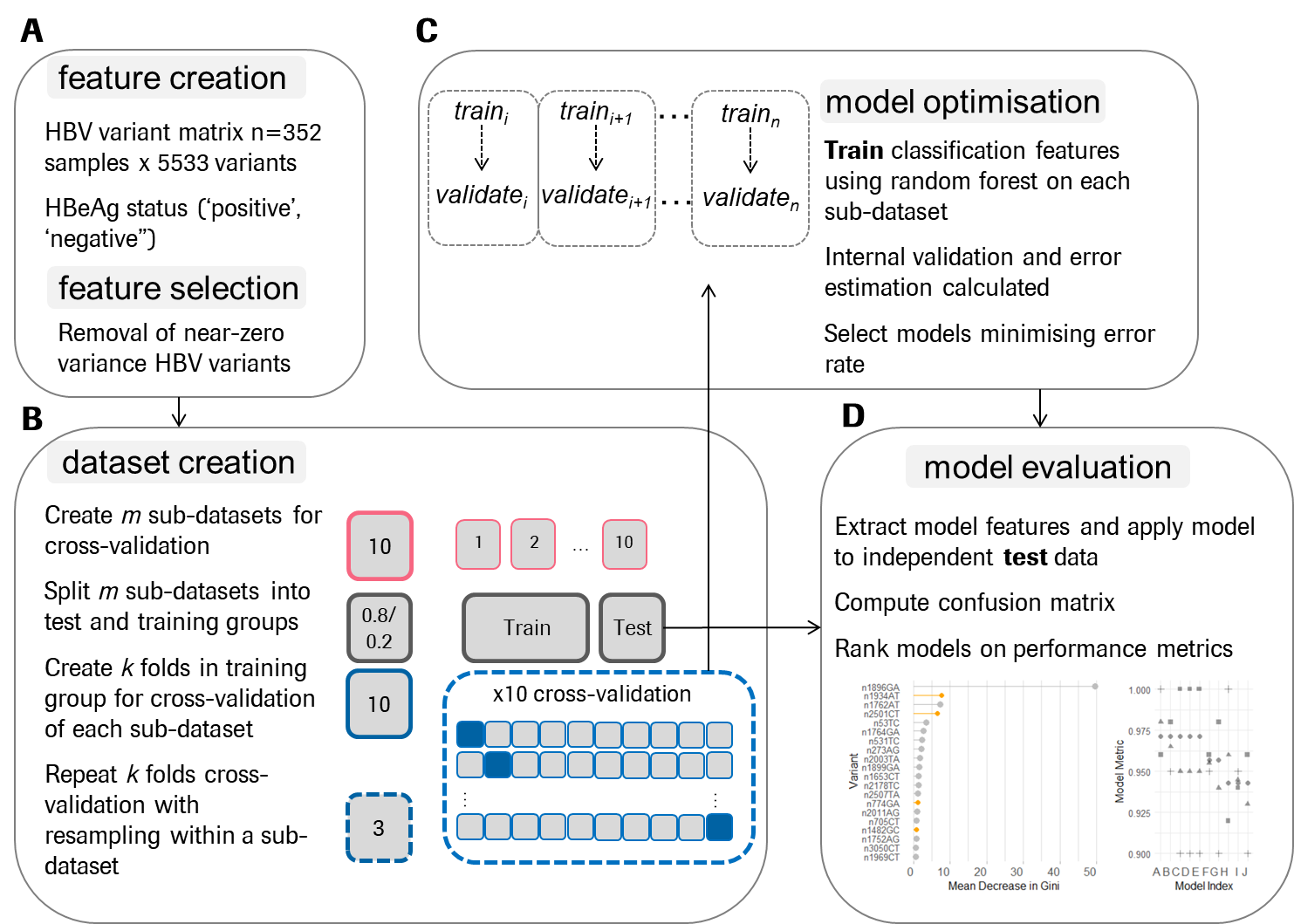


**Supplementary Figure 3**: Schematic of machine learning approach for combined datasets. **A** – Feature creation: Features are variants found at a frequency of 1% of the quasispecies population. Datasets A and B are combined to create a matrix of n=352 samples from patient plasma with a total of n=5533 unique variants. The HBeAg status for each patient was defined as ‘positive’ or ‘negative’. Feature selection consisted of the removal of variants with near-zero variance (n=432 remaining). **B** – Dataset creation: the combined matrix was divided into ten sub-datasets using balanced partitioning such that positive and negative samples were represented in each sub-dataset in the proportions found in the whole dataset. These sub-datasets were further partitioned into test and training sets in a 0.2:0.8 or 0.1:0.9 ratio. Each training set from each sub-dataset divided into ten folds for internal cross-validation. **C** – Model optimization: a random forest model was optimized for each training set using three repeats of the 10-fold cross validation by minimizing the estimated error rate. This was based on a maximum of 500 trees. Ten consensus models were returned and the best performing models from each run were selected. **D** – Model evaluation: optimised models were tested against the appropriate test datasets. A confusion matrix was used to define the model metrics based upon the accuracy of prediction of positive and negative status. Models were ranked on performance metrics and the model with the highest balanced accuracy was presented.

**
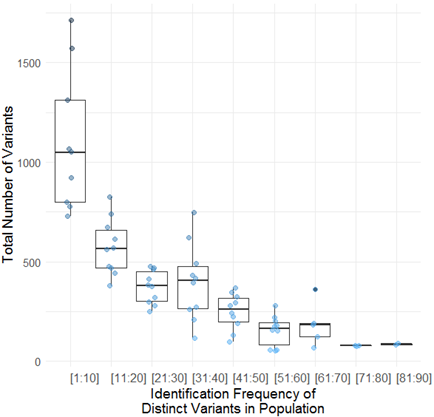
**

**Supplementary Figure 4**: The majority of variants in Dataset A are found in fewer than ten patient samples (box-and-whisker plot; data points represent individual patients) indicating that the majority of viral quasispecies are distinct to each patient. In a box-and-whisker plot the box represents the interquartile range; whiskers show the maximum and minimum values; the central horizontal line shows the median. Plots are binned for multiples of ten patients.


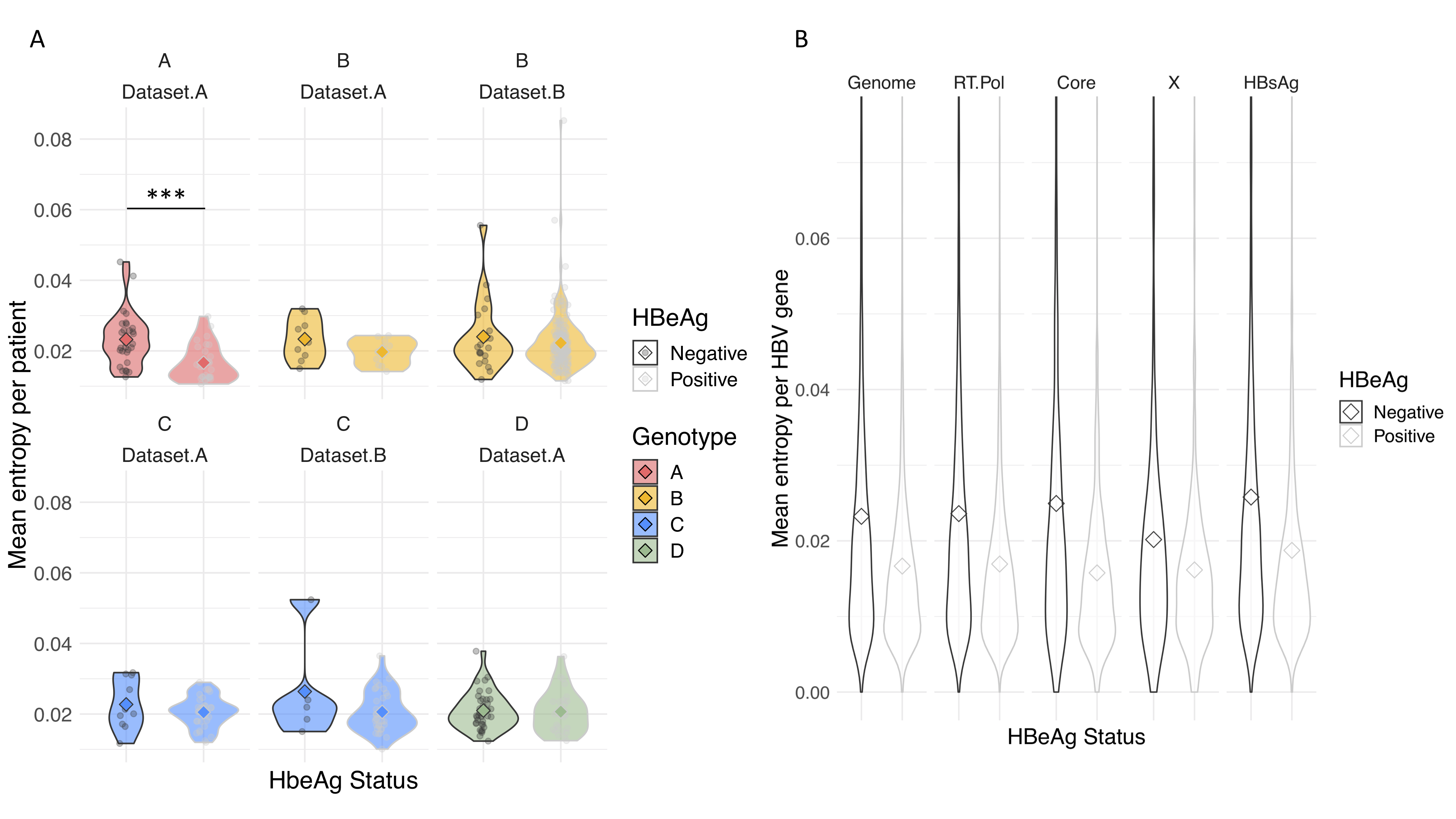


**Supplementary Figure 5: A:** Violin plots of mean whole HBV genome entropy *per* patient plotted by genotype and HBeAg status – data points represent patient means; diamond defines the overall mean. Negative HBeAg status is represented by dark borders; positive HBeAg status is represented by pale grey borders. When entropy is equal to zero the probability of an outcome occurring is certain. A significant difference in entropy is found between positive and negative HBeAg status for genotype A (p=2.8e-04), but not for other genotypes when considered across all patients; **B**: This difference in genotype A was also evident when entropy was considered per gene (p<0.001 for all comparisons). Plot y-axis are reset to highlight interquartile range. Significant differences in entropy were also seen between HBeAg status for genes in genotype B, but not for C or D (Supplementary Figure 5). Genotype E is not considered.


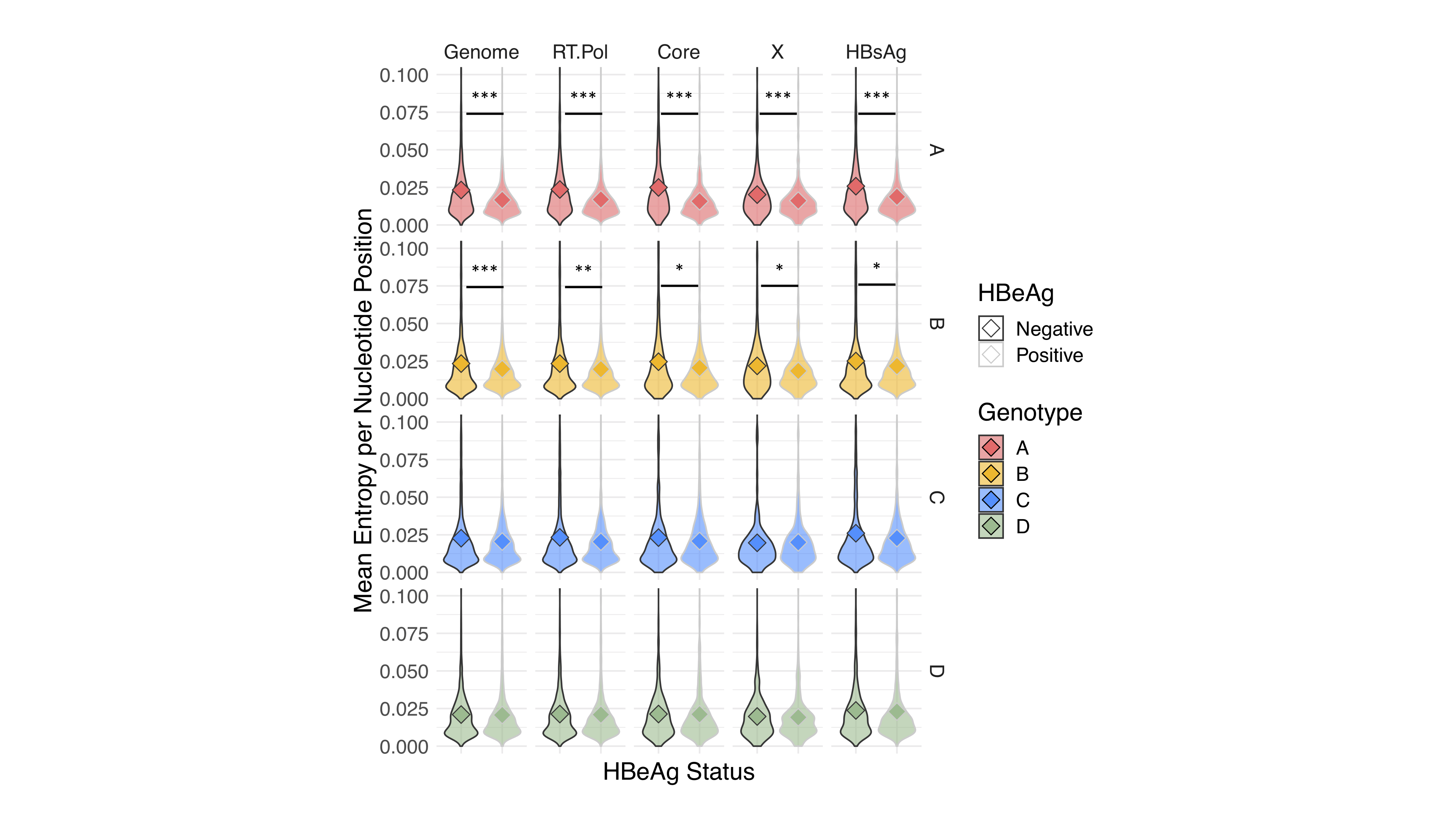


**Supplementary Figure 6:** Violin plots of entropy per gene or across the whole genome for HBV genotypes A-D derived from Dataset A – distribution of mean entropy per nucleotide position; diamond defines the overall mean. Statistical comparisons are made between the mean entropy for HBV HBeAg status in each context (Negative- dark border, Positive – light border). Results of hypothesis testing: ‘***’ - <0.001; ‘**’ - <0.01; ‘*’ - <0.05. When entropy is equal to zero the probability of an outcome occurring is certain. Significant differences exist between the mean entropy per gene, and across the whole genome, for Genotype A with HBeAg negative status consistently demonstrating an increased probability of disorder at the nucleotide level (lower entropy), i.e. within each gene in HBeAg negative samples the probability of observing a specific event is reduced or change at a nucleotide position is increased. In genotype B differences are evident, especially across the whole genome, but no differences were evident between groups for genotypes C and D.

**
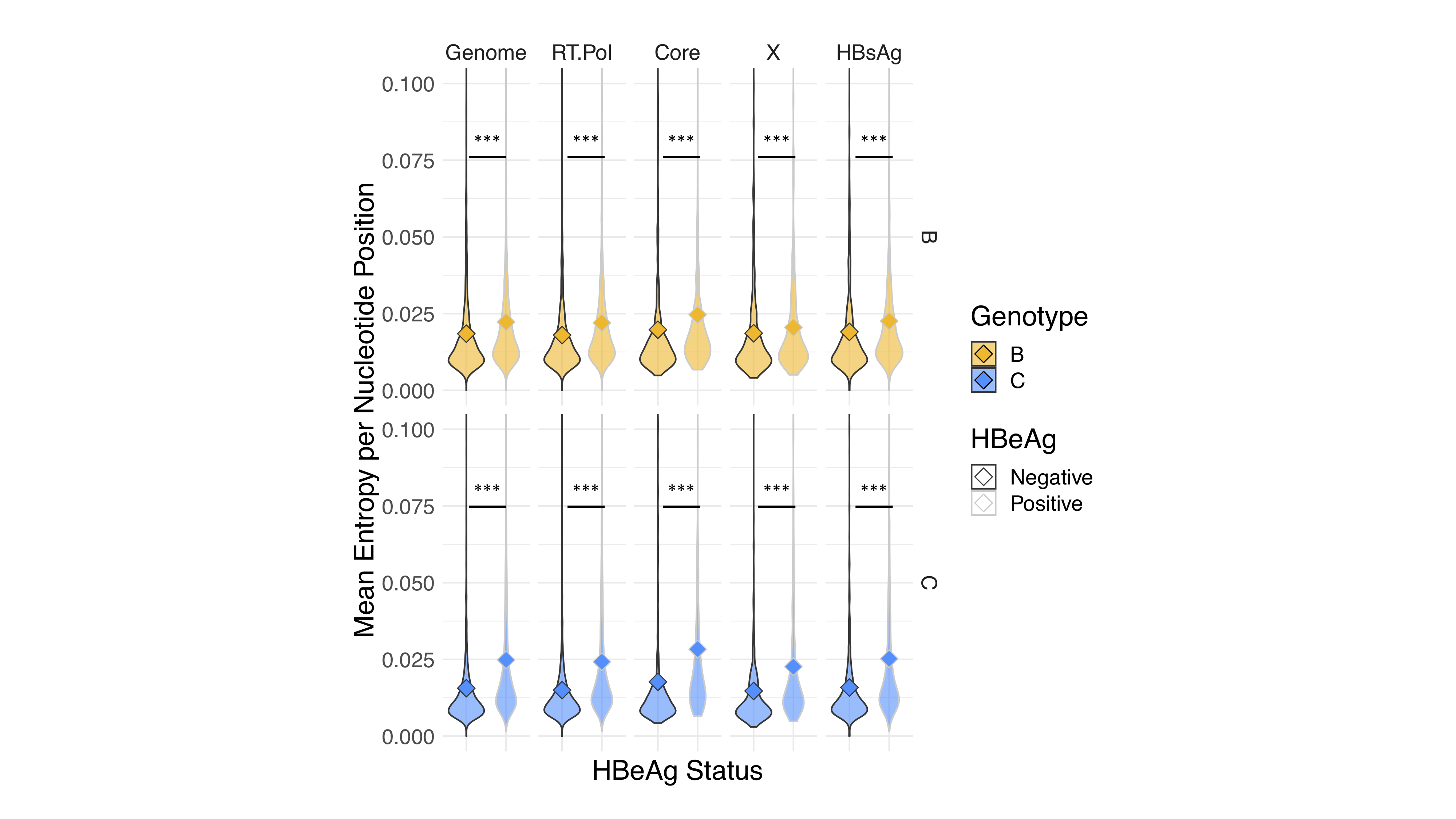
**

**Supplementary Figures 7:** Violin plots of entropy per gene or across the whole genome for HBV genotypes B and C derived from Dataset B– distribution of mean entropy per nucleotide position; diamond defines the mean. Statistical comparisons are made between the mean entropy for HBV HBeAg status in each context (Negative- dark border, Positive – light border). Results of hypothesis testing: ‘***’ - <0.001; ‘**’ - <0.01; ‘*’ - <0.05. In contrast to Dataset A the findings in Dataset B demonstrated higher entropy in HBeAg positive patients (p<0.001 for all comparisons).

**Supplementary Tables**

| **Primer** | **Position** | **Sequence (5’~3’)** | **Amplicon name** | **Amplicon Length** |
| --- | --- | --- | --- | --- |
| Primer F1-F | 1823~1845 | TTTTTCACCTCTGCCTARTCATC | FI | 3.2kb |
| Primer F1-R | 1808~1827 | AAAAAGTTGCATGGTGCTGG |  |  |
| Primer S1-F | 1609~1627 | CATGGARACCACCGTGAAC | S1 | 676 nt |
| Primer S1-R | 2263~2284 | TGCGAATCCACACTCCRAAAGA |  |  |
| Primer SR1-F | 247~269 | AGTCTAGACTCGTGGTGGACTTC | SR1 | 871 nt |
| Primer SR1-R | 1095~1117 | GCCTTRTAAGTTGRCGAGAAAGT |  |  |
| Primer SR2-F | 968~988 | CAGRCCTATTGATTGGAAAGT | SR2 | 834 nt |
| Primer SR2-R | 1782~1801 | GACCAATTTRTGCCTACAGC |  |  |
| Primer SR3-F | 2062~2081 | GCACTCAGGCAAGCHATTCT | SR3 | 772 nt |
| Primer SR3-R | 2808~2833 | CCAAGAATATGGTGACCCRCAAAATG |  |  |
| Primer SR4-F | 2639~2658 | ATGCCTGCTAGRTTYTATCC | SR4 | 932 nt |
| Primer SR4-R | 370~388 | GCAGACACATCCAGCGATA |  |  |

**Supplementary Table 1**: Primers for the amplification of whole genome or fragments for Dataset B protocol.

| **ID** | **POS** | **REF_1** | **REF_2** | **ALT_1** | **ALT_2** | **AF_1** | **AF_2** | **DIFF** | **Genotype** |
| --- | --- | --- | --- | --- | --- | --- | --- | --- | --- |
| Patient2 | 1838 | A | NA | G | NA | 0.396623 | NA | 0.3966 | A |
| Patient3 | 1638 | C | C | T | T | 0.92925 | 0.724913 | 0.20434 | A |
| Patient3 | 1674 | T | T | C | C | 0.93242 | 0.72378 | 0.20864 | A |
| Patient3 | 2869 | T | T | G | G | 0.941916 | 0.729475 | 0.21244 | A |
| Patient9 | 783 | G | G | A | A | 0.663647 | 0.420877 | 0.24277 | C |
| Patient9 | 1991 | C | C | A | A | 0.694308 | 0.382663 | 0.31165 | C |
| Patient9 | 2465 | T | T | A | A | 0.806019 | 0.392708 | 0.41331 | C |
| Patient9 | 3051 | C | C | T | T | 0.794183 | 0.493086 | 0.3011 | C |

| **ID** | **POS** | **REF_1** | **REF_2** | **ALT_1** | **ALT_2** | **AF_1** | **AF_2** | **DIFF** | **Genotype** |
| --- | --- | --- | --- | --- | --- | --- | --- | --- | --- |
| Patient1 | 2088 | G | G | T | T | 0.467924 | 0.722546 | -0.25462 | A |
| Patient10 | 2870 | G | G | C | C | 0.411135 | 0.692251 | -0.28112 | D |
| Patient10 | 2895 | C | C | A | A | 0.478835 | 0.707401 | -0.22857 | D |
| Patient4 | 587 | G | G | A | A | 0.248032 | 0.473093 | -0.22506 | C |
| Patient6 | 1764 | G | G | A | A | 0.232818 | 0.722 | -0.48918 | C |
| Patient9 | 2804 | T | T | C | C | 0.198795 | 0.577431 | -0.37864 | C |
| Patient9 | 544 | A | A | C | C | 0.168694 | 0.531697 | -0.363 | C |
| Patient9 | 3051 | C | C | A | A | 0.201023 | 0.494063 | -0.29304 | C |
| Patient9 | 60 | C | C | T | T | 0.163574 | 0.455846 | -0.29227 | C |
| Patient9 | 2793 | A | A | T | T | 0.152526 | 0.444607 | -0.29208 | C |
| Patient9 | 2352 | G | G | C | C | 0.181659 | 0.465578 | -0.28392 | C |
| Patient9 | 2079 | T | T | G | G | 0.154617 | 0.43719 | -0.28257 | C |
| Patient9 | 2080 | G | G | T | T | 0.152978 | 0.432116 | -0.27914 | C |
| Patient9 | 2138 | G | G | T | T | 0.101772 | 0.365891 | -0.26412 | C |
| Patient9 | 453 | A | A | G | G | 0.453673 | 0.71314 | -0.25947 | C |
| Patient9 | 783 | G | G | T | T | 0.239597 | 0.491125 | -0.25153 | C |
| Patient9 | 1068 | A | A | C | C | 0.118001 | 0.347682 | -0.22968 | C |
| Patient9 | 3014 | A | A | G | G | 0.111013 | 0.340035 | -0.22902 | C |

**Supplementary Table 2a and 2b**: Table 2a shows variants with >20 percentage point difference between plasma and liver allele frequencies; Table 2b shows variants with >20 percentage point difference between liver and plasma allele frequencies. Patient 9 shows the greatest differential between liver and plasma variant frequencies. Column names: ‘ID’: patient identifier; ‘POS’: genome position; ‘REF_1’ - plasma; ‘REF_2’ – liver reference, ALT_1 and ALT_2 – alternative alleles for plasma and liver respectively; AF_1 and AF_2: allele frequencies for plasma and liver respectively; DIFF – allele frequency difference plasma vs. liver; Genotype – genotype defined for sample.

| Dataset | *Minimum* | *Mean* | *Maximum* |
| --- | --- | --- | --- |
| A | 161,010 | 477,118 | 1,051,936 |
| A.filtered | 146,693 | 440,806 | 987,702 |
| B | 389,661 | 961,260 | 1,964,386 |
| B.filtered | 364,737 | 894,280 | 1,785,635 |

**Supplementary Table 3**: Summary of average number of reads per sample, with range, before, and after, filtering.

**References**

[1] Lassmann T, Sonnhammer EL. Kalign – an accurate and fast multiple sequence alignment algorithm. BMC Bioinformatics 2005;6:298. doi:10.1186/1471-2105-6-298.

[2] Remita MA, Halioui A, Malick Diouara AA, Daigle B, Kiani G, Diallo AB. A machine learning approach for viral genome classification. BMC Bioinformatics 2017;18:208. doi:10.1186/s12859-017-1602-3.

[3] Schliep KP. phangorn: phylogenetic analysis in R. Bioinformatics 2011;27:592–3. doi:10.1093/bioinformatics/btq706.

[4] Wickham H. Ggplot2: Elegant Graphics for Data Analysis. 2nd ed. Springer Publishing Company, Incorporated; 2009.

[5] Yu G, Smith DK, Zhu H, Guan Y, Lam TT-Y. ggtree: an r package for visualization and annotation of phylogenetic trees with their covariates and other associated data. Methods Ecol Evol 2017;8:28–36. doi:10.1111/2041-210X.12628.

[6] Shannon, C.SE. A Mathematical Theory of Communication. Bell Syst Tech J 1948;27:379–423.

[7] Nishijima N, Marusawa H, Ueda Y, Takahashi K, Nasu A, Osaki Y, et al. Dynamics of Hepatitis B Virus Quasispecies in Association with Nucleos(t)ide Analogue Treatment Determined by Ultra-Deep Sequencing. PLOS ONE 2012;7:e35052. doi:10.1371/journal.pone.0035052.

[8] Bolger AM, Lohse M, Usadel B. Trimmomatic: a flexible trimmer for Illumina sequence data. Bioinformatics 2014;30:2114–20. doi:10.1093/bioinformatics/btu170.

[9] Li H, Handsaker B, Wysoker A, Fennell T, Ruan J, Homer N, et al. The Sequence Alignment/Map format and SAMtools. Bioinformatics 2009;25:2078–9. doi:10.1093/bioinformatics/btp352.

[10] Okonechnikov K, Conesa A, García-Alcalde F. Qualimap 2: advanced multi-sample quality control for high-throughput sequencing data. Bioinformatics 2016;32:292–4. doi:10.1093/bioinformatics/btv566.

[11] Posada-Cespedes S, Seifert D, Beerenwinkel N. Recent advances in inferring viral diversity from high-throughput sequencing data. Virus Res 2017;239:17–32. doi:10.1016/j.virusres.2016.09.016.

[12] Verbist BMP, Thys K, Reumers J, Wetzels Y, Van der Borght K, Talloen W, et al. VirVarSeq: a low-frequency virus variant detection pipeline for Illumina sequencing using adaptive base-calling accuracy filtering. Bioinforma Oxf Engl 2015;31:94–101. doi:10.1093/bioinformatics/btu587.

[13] Wilm A, Aw PPK, Bertrand D, Yeo GHT, Ong SH, Wong CH, et al. LoFreq: a sequence-quality aware, ultra-sensitive variant caller for uncovering cell-population heterogeneity from high-throughput sequencing datasets. Nucleic Acids Res 2012;40:11189–201. doi:10.1093/nar/gks918.

[14] Cingolani P, Platts A, Wang LL, Coon M, Nguyen T, Wang L, et al. A program for annotating and predicting the effects of single nucleotide polymorphisms, SnpEff. Fly (Austin) 2012;6:80–92. doi:10.4161/fly.19695.

[15] Kuhn M. Building Predictive Models in R Using the caret Package. J Stat Softw Vol 1 Issue 5 2008 2008.

[16] Wang M, Zhao Y, Zhang B. Efficient Test and Visualization of Multi-Set Intersections. Sci Rep 2015;5:16923. doi:10.1038/srep16923.

[17] Krzywinski MI, Schein JE, Birol I, Connors J, Gascoyne R, Horsman D, et al. Circos: An information aesthetic for comparative genomics. Genome Res 2009. doi:10.1101/gr.092759.109.
